# Intersection of Diet and Exercise with the Gut Microbiome and Circulating Metabolites in Male Bodybuilders: A Pilot Study

**DOI:** 10.3390/metabo12100911

**Published:** 2022-09-27

**Authors:** Alison W. S. Luk, Lachlan Mitchell, Yen Chin Koay, John F. O’Sullivan, Helen O’Connor, Daniel A. Hackett, Andrew Holmes

**Affiliations:** 1 Charles Perkins Centre, The University of Sydney, Camperdown, NSW 2006, Australia; 2 School of Life and Environmental Sciences, The University of Sydney, Camperdown, NSW 2006, Australia; 3 Exercise, Health and Performance, School of Health Sciences, Faculty of Medicine and Health Sciences, The University of Sydney, Camperdown, NSW 2006, Australia; 4 Heart Research Institute, The University of Sydney, Newtown, NSW 2042, Australia; 5 Department of Cardiology, Royal Prince Alfred Hospital, Camperdown, NSW 2050, Australia

**Keywords:** personalised diet, athletes, gut microbiota, macronutrient ratio

## Abstract

Diet, exercise and the gut microbiome are all factors recognised to be significant contributors to cardiometabolic health. However, diet and exercise interventions to modify the gut microbiota to improve health are limited by poor understanding of the interactions between them. In this pilot study, we explored diet–exercise–microbiome dynamics in bodybuilders as they represent a distinctive group that typically employ well-defined dietary strategies and exercise regimes to alter their body composition. We performed longitudinal characterisation of diet, exercise, the faecal microbial community composition and serum metabolites in five bodybuilders during competition preparation and post-competition. All participants reduced fat mass while conserving lean mass during competition preparation, corresponding with dietary energy intake and exercise load, respectively. There was individual variability in food choices that aligned to individualised gut microbial community compositions throughout the study. However, there was a common shift from a high protein, low carbohydrate diet during pre-competition to a more macronutrient-balanced diet post-competition, which was associated with similar changes in the gut microbial diversity across participants. The circulating metabolite profiles also reflected individuality, but a subset of metabolites relating to lipid metabolism distinguished between pre- and post-competition. Changes in the gut microbiome and circulating metabolome were distinct for each individual, but showed common patterns. We conclude that further longitudinal studies will have greater potential than cross-sectional studies in informing personalisation of diet and exercise regimes to enhance exercise outcomes and improve health.

## 1. Introduction

Non-communicable diseases are the leading causes of death worldwide, with cardiovascular disease accounting for around half of non-communicable disease deaths [1]. Modifiable lifestyle factors, including an imbalanced diet and a lack of physical activity, are major contributors to cardiovascular disease risk [2]. However, the optimum diet and exercise regime remains elusive, and diet and exercise interventions often have highly variable outcomes between individuals. This is evident in sports science, where defined diet strategies have been designed to optimise energy availability and complement athletic outcomes [3]. Yet, individual factors are the main determinant of athletic performance. In recent years, with the recognition of the significance of the gut microbiome in modulating metabolism, there has been an increasing interest in the contribution of the gut microbiome to individuality in athletic outcomes [4,5,6].

The impact of diet on human physiology and metabolism is complex and occurs through mechanisms that are dependent on the human digestive processes and microbial activity. Although most nutrients are directly released from food by digestive processes and absorbed in the small intestine, the ileal and colonic gut microbes profoundly influence the outcomes. Microbial metabolism results in the formation or modification of a wide range of molecules, including nutrients (e.g., amino acids, vitamins, short-chain fatty acids) and other bioactive molecules (e.g., secondary bile acids, polyphenols) [7]. Collectively, these microbial metabolites account for a significant proportion of energy (>10%), essential nutrient supply, and contribute to the signaling interactions that underpin physiological regulation [7,8]. While dietary guidelines aimed at altering body composition often outline specific macronutrient compositions and energy intakes [9], there is flexibility in the choice of food items. Thus the chemical composition of different food drive distinct interactions with the gut microbes [10,11,12]. Hence, to understand the outcomes of diet manipulation, dietary analysis needs to be considered at multiple levels (e.g., nutrients, foods, meals, diets) [13]. Furthermore, the microbial contribution to the shaping of diet-derived molecules also needs to be considered [14].

Recent studies provide evidence that gut microbial metabolites influence the effects of exercise on physiological outcomes. Germ-free mice, devoid of a microbiota, had reduced muscle mass, fat mass, and exercise endurance compared to conventionally raised mice with a microbiota [15]. In another mouse study, intrarectal instillation of the microbial metabolite propionate compared to a control of saline increased exercise performance [16]. Interestingly, another study found that although exercise training of mice altered the microbiota composition and diversity, the protective metabolic effects of exercise were not mediated through gut metabolites (as tested by faecal microbiota transfer), but through diabetogenic diet effects [17]. Such studies highlight that diet, exercise and microbes interact, however, identifying these relationships in humans is challenging.

There have been reports of specific microbial taxa being associated with exercise outcomes. For example, in cyclists a greater exercise load was associated with a higher relative abundance of *Prevotella* spp. [18], while marathon athletes were reported to have a higher relative abundance of *Veillonella* spp. in the five days post-marathon compared to pre-marathon [16]. However, recent reviews indicated that there were no specific microbial taxa, or community composition consistently associated with exercise regimes or athletic performance level [4,5,6]. Furthermore, while studies found differences between the microbiotas of athletes and sedentary individuals, the variation between individuals was often greater [19,20,21]. Together, these studies indicate that exercise-microbiome relationships are complex. The duration of exercise [22] and the type of sport [23] have different impacts on the gut microbiota. Factors other than exercise also need to be considered. In a study of endurance runners, athletic performance was greater with a high carbohydrate diet intervention compared to a high protein diet, but also was also associated with individuals whose microbiota had greater resistant to change during the diet intervention [24]. In summary, while diet-derived microbial metabolites are known to impact human physiology and metabolism [25,26], the influence of exercise on the gut microbiome and vice versa remains poorly predictable.

In our study, we sought the understand how variations between individuals impacted athletic outcomes. Bodybuilders are a group of athletes well-suited for investigating the dynamics between diet, exercise, and the gut microbiome, since they typically undergo strongly patterned dietary manipulation and exercise to modulate their body composition for a competition [27,28,29]. Bodybuilders usually begin preparation 20 weeks prior to the competition, using exercise and dietary strategies to reduce fat mass to increase muscle definition while limiting loss of muscle mass [29]. During competition preparation, bodybuilder’s diets are characterised by a high protein intake of >1.9 g/kg/d to maintain lean mass [27], combined with progressive reductions in carbohydrate, fat, and total energy intake to create an energy deficit to achieve fat loss [30]. After the competition, bodybuilders tend to have a more relaxed diet resulting in an increase in energy intake, with some bodybuilders reporting episodes of overindulgent eating [30]. This dietary pattern means that bodybuilders are predicted to undergo transitions in the ratio of protein to carbohydrate in the diet at levels that are significant drivers of microbial community dynamics [31].

In this pilot study of five male bodybuilders, we tested the hypothesis that defined transitions in diet composition and exercise training are associated with changes in the gut microbial community composition and circulating metabolites. We performed a longitudinal investigation, sampling at eight weeks and one week prior the competition and four weeks post-competition to determine diet- and exercise-associated effects on the microbiome and host metabolism. We aimed to explore the deviations between participants to better understand the impact of individual differences on diet and exercise outcomes.

## 2. Materials and Methods

### 2.1. Participants

The study was conducted in accordance with the Declaration of Helsinki, and approved by the Human Ethics Committee of THE UNIVERSITY OF SYDNEY (protocol code 2015/425, date of approval: 7 July 2015). Informed consent was obtained from all subjects involved in the study.

This study is based on a subset of competitive bodybuilders from a previously reported study [32]. The inclusion criteria were: male, aged 18 years or above, drug-free, and preparing to compete in a natural federation bodybuilding competition. For this study we selected five participants with a longitudinal series of blood and faeces samples matched with diet and exercise history to look for associations with the gut microbiota and circulating metabolites over time. These five bodybuilders had an average of 28.0 ± 11.9 years of age, 177.0 ± 2.8 cm height, 77.7 ± 6.7 kg body weight, and 4.2 ± 3.5 years bodybuilding experience.

Participants were assessed on three occasions over a 12-week period. Samples were collected eight weeks and one week prior to competition (PRE8, PRE1), and four weeks after the competition (POST4). Participants presented to the laboratory for blood sampling between 0600 and 0800 h after a 12 h food and fluid fast. Participants were also instructed to abstain from caffeine, alcohol and exercise in the 12 h prior to the blood test.

### 2.2. Blood Sampling and Serum Metabolite Analysis

Venous blood samples were obtained by venepuncture from the antecubital vein. Samples were centrifuged after allowing 15 min for clotting, and serum separated and stored at −80 °C until analysis. 10 µL aliquots of serum samples were prepared via protein precipitation with the addition of nine volumes of 74.9:24.9:0.2 *v*/*v*/*v* acetonitrile/methanol/formic acid containing stable isotope-labelled internal standards: valine-d_8_ (Sigma-Aldrich, St. Louis, MO, USA), and phenylalanine-d_8_ (Cambridge Isotope Laboratories, Tewksbury, MA, USA).

Targeted metabolomic analysis measured hydrophilic metabolites in positive and negative ionisation mode using an LC-MS system comprised of an Agilent 1260 Infinity liquid chromatography (LC) system coupled to a QTRAP 5500 mass spectrometer (MS) (AB Sciex, Foster City, CA, USA). A hydrophilic interaction liquid chromatography (HILIC)—tandem mass spectrometry (LC-MS/MS) method was used for the simultaneous detection of polar metabolites in positive ionisation mode, composed of amino acids, nucleotides, neurotransmitters and vitamins [33,34]. An amide chromatographic LC-MS/MS method was used for the detection of nucleotide and nucleoside phosphates, high-energy intermediates, organic acids, Krebs cycle intermediates, and glycolytic intermediates [35]. For each method, quality control (QC) pooled serum samples were included in the analytical run spaced at regular intervals of every five injections, enabling monitoring and correction for temporal drift in mass spectrometry performance. All raw data files from Analyst software v1.6.2 (AB Sciex) were imported into Multi-Quant v3.0 (AB Sciex) for MRM Q1/Q3 peak integration and the abundance of each metabolite in each sample was normalised to the nearest neighbour flanking pair of pooled serum, deriving a normalised area (AU) for each metabolite.

### 2.3. Diet and Exercise

Weighed food and training diaries were completed by participants during the seven days before each assessment time point. The food diaries documented all food, fluid, and supplements consumed. Food diaries were analysed using FoodWorks v8 (Xyris Software, Brisbane, Australia) and included analysis of reported dietary supplement consumption. The training diaries documented aerobic and resistance training that the participants performed. Body composition, exercise training, energy intake and macronutrient intake in the seven days prior each timepoint is shown in Table S1. Supplement intake in the seven days prior to each timepoint is shown in Table S2.

### 2.4. Dual-Energy X-ray Absorptiometry (DXA)

Body composition was estimated using the Lunar Prodigy whole body DXA scanner (GE Healthcare, Chicago, IL, USA) as previously reported [32]. Total fat mass and lean mass were determined using enCORE 2011 v13.60.033 (GE Healthcare).

### 2.5. Faecal Sampling and Microbial Analysis

Participants were provided with 70 mL stool collection containers (TechnoPlas, St Marys, Australia), and instructed to collect and store the specimen at home. For each participant, one sample was self-collected within the seven days prior to each timepoint (the same period as when the food diaries were documented). Samples were collected using the provided container, and immediately frozen at home without preservatives, before being returned to the laboratory within seven days and stored at −80 °C until analysis. The faeces samples were thawed immediately prior to DNA extraction. Around 500 mg of faeces from the centre of the sample was isolated for DNA extraction.

Total DNA was extracted from faecal samples using the FastDNA SPIN kit for feces (MP Biomedicals, Santa Ana, CA, USA). The DNA extraction protocol was modified from the manufacturer’s instructions by: repeating the homogenisation step once, repeating the wash step with wash buffer #2 twice, and extending the centrifugation step to extract residual ethanol to 4 min.

The microbial community was profiled using the 16S rRNA V4 region (515F–806R) [36]. Sequencing with Illumina MiSeq was performed at the Ramaciotti Centre for Genomics (University of NSW, Sydney, Australia). Paired-end reads were aligned using Pandaseq [37]. Chimera checking was performed with Usearch v10.0.240 [38]. Sequenced reads were assigned to operational taxonomic units (OTUs) at 97% identity with open reference picking against the GreenGenes v13.8 database using the QIIME v1.9.1 pipeline [39]. OTUs that did not reach at least 0.1% abundance in at least three samples were filtered out to reduce noise in the dataset. After filtering, the minimum reads per sample was 80,000.

The microbial raw sequence reads presented in this study are openly available in the National Center for Biotechnology Information (NCBI) Sequence Read Archive, accession numbers SRR10317043-SRR10317057.

### 2.6. Statistical Analysis

The relative changes in body composition and exercise training at the PRE1 and POST4 timepoints were compared to the PRE8 timepoint as a baseline.

Statistical analysis of serum metabolites was performed with IBM SPSS Statistics v24.0.0.0 (IBM, Armonk, NY, USA). The differences in metabolite levels between timepoints were analysed using the nonparametric Kruskal–Wallis test, with a significance level set at *p* < 0.05. The sample for participant 5 at the PRE1 timepoint did not pass LC-MS quality control and was excluded from analysis.

Principal component analyses of dietary food components and serum metabolites were performed in R with the stats package v3.6.1 [40] and visualised with the ggbiplot package v0.55 [41].

Microbial diversity and principal coordinate analysis was performed in R [40] with the phyloseq package v1.26.1 [42] and visualised with the ggplot2 package v3.1.0 [43]. Within-sample diversity was measured using the inverse Simpson index and between-sample diversity was measured using the weighted UniFrac metric. Permutational multivariate analysis of variance of weighted UniFrac distances was performed using the vegan package v2.5.4 [44].

## 3. Results

### 3.1. Bodybuilders Showed a Common Direction of Change in Body Composition Alteration but Had Varied Exercise Regimes

**Figure 1:**
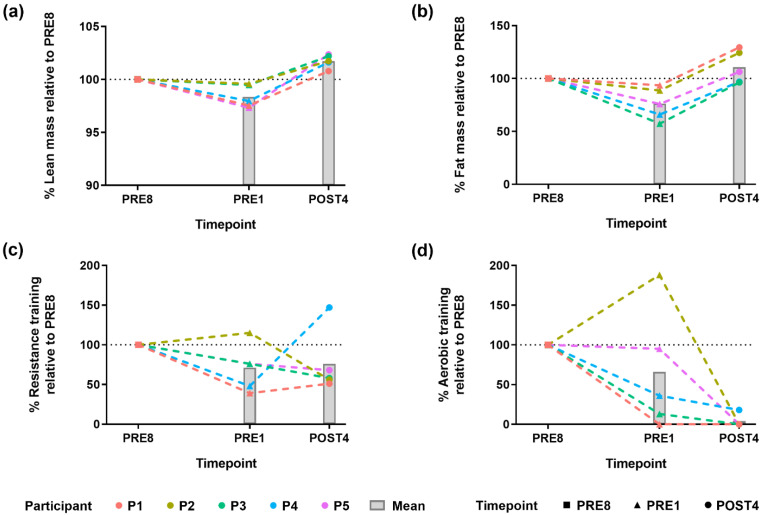
General and individual changes in body composition alteration and exercise training: (**a**,**b**) Body composition relative to the PRE8 timepoint of each participant; (**c**,**d**) Exercise training relative to the PRE8 timepoint of each participant; Dotted lines represent each participant, bars represent mean; n = 5 participants; Absolute values for body composition and exercise training are shown in Table S1.

We investigated common patterns and individual deviations of body composition alteration and exercise training across bodybuilders during the pre- and post-competition periods. All participants achieved intended body composition change over the competition preparation period (PRE8 to PRE1 timepoint), with a greater reduction in fat mass (6.4 to 43% reduction) than loss in lean mass (0.4 to 2.7% reduction) (Figure 1a,b, Table S1). Outcomes for the participants were not equal, with participants 2 and 3 being more successful in conserving lean mass (<0.5% reduction) than others. However, participant 3 had a much greater reduction in fat mass than participant 2 (43% compared to 11% reduction). Participant 1 was distinctive with the least fat mass reduction (6.4% reduction). After the competition (PRE1 to POST4 timepoint), all participants increased both lean mass and fat mass.

Exercise training reduced on average at PRE1 and POST4 compared to PRE8, but the exercise regimes varied across individuals (Figure 1c,d). Notably, an increase in both resistance and aerobic training from PRE8 to PRE1, as only observed in participant 2, corresponded with better outcomes in the conservation of lean mass, but not with loss of fat mass. Despite our small sample size, it is evident that exercise alone does not explain the extent of body composition alteration.

### 3.2. Bodybuilders Diets Were Similar at the Energy and Macronutrient Level but Variable at the Food Item Level

**Figure 2:**
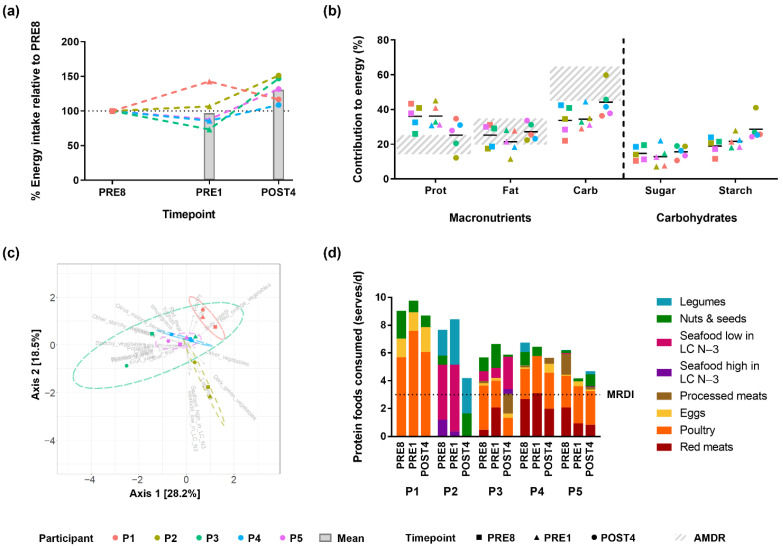
Dietary intake patterns at different levels: (**a**) Energy intake relative to the PRE8 timepoint of each participant, inclusive of diet and supplements (Table S2); Dotted lines represent each participant; Bars represent mean (n = 5 participants); (**b**) Contribution of macronutrients and carbohydrate types to energy intake; Black lines represent mean (n = 5 participants); Striped bars represent the acceptable macronutrient distribution range (AMDR) [45]; (**c**) Principal component analysis of food items; Vectors indicate the contribution of each food item to the diet; Ellipses indicate 95% confidence intervals of samples from each participant; Please see Figure S2 for a larger version of (**c**); (**d**) Serves of protein foods consumed as compared to the minimum recommended daily intake (MRDI) [46]; LC N-3 = long chain n-3 fatty acids; Serves of other food types are shown in Figure S1. Supplement intake is shown in Table S2.

We then investigated dietary intake at three levels of complexity: energy, macronutrients, and food items. At the level of energy intake, participants showed similar patterns. Energy intake was highest after the competition in four out of five participants (Figure 2a). Notably, greater decreases in energy intake from PRE8 to PRE1 corresponded to greater reductions in fat mass from PRE8 to PRE1 (Figure 1b) but was not linked to changes in lean mass.

All participants showed a similar shift in macronutrient intake across timepoints with respect to the acceptable macronutrient distribution range (AMDR) guidelines [45] (Figure 2b). Prior the competition, protein contribution to energy was consistently above the AMDR upper limit (>25%), carbohydrate contribution to energy was below the AMDR lower limit (<45%), while energy intake as fat was generally within the AMDR (20 to 35%). After the competition, the dietary macronutrient distribution of all participants shifted towards the AMDR. This was attributed to an increase in starch intake.

All participants exceeded the minimum recommended daily intake (MRDI) [46] for protein foods at all timepoints, and showed variation at the level of food items (Figure 2c,d, Figure S1). Participants 4 and 5 consumed similar food items, as shown by close clustering of their diets in principal components analysis. Comparatively, the diets of participant 1 and 2 each formed distinct clusters away from other participants, attributed to the differences in their choice of protein foods (Figure 2d). The diet of participant 1 was characterised by a high intake of poultry and eggs, while participant 2 had a high intake of legumes. Participants also showed high variability in the consumption of supplements containing protein or amino acids (Table S2). However, there was no clear pattern between the intake of these supplements alone and body composition alteration.

In summary, at the level of energy and macronutrient intake there were pronounced patterns of similarity across participants, while at the level of food items there was interindividual variation.

### 3.3. Bodybuilders Maintained Individualised Gut Microbial Communities with Altered Diversity Post-Competition

**Figure 3:**
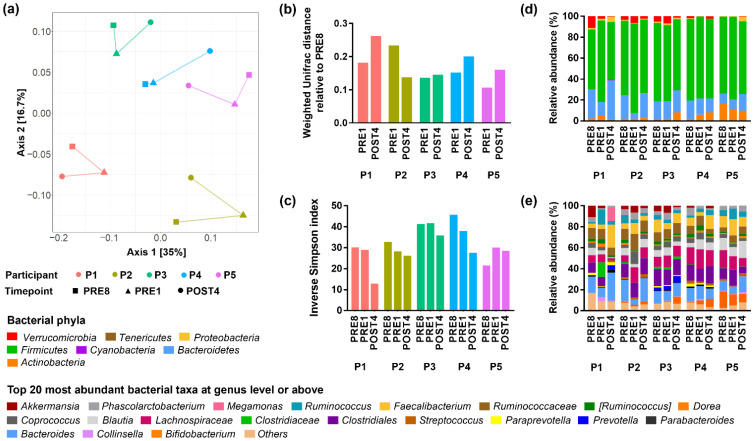
Shifts in gut microbial community composition within and between individuals: (**a**) Principal coordinate analysis ordination of weighted UniFrac distances; Connecting lines indicate shifts in microbial community composition across timepoints; (**b**) Weighted UniFrac distances relative to the PRE8 timepoint; Higher values indicate greater between-sample diversity; (**c**) Inverse Simpson index; Higher values indicate greater within-sample diversity; (**d**) Relative abundance of bacteria taxa grouped at phylum level; (**e**) Relative abundance of bacteria taxa grouped at genus level or the next lowest taxonomic assignment; [Square brackets] around taxa indicates uncertain taxon assignation.

We next looked for patterns in microbiota responses over time and across participants. Each participant had an individualised and dynamic gut microbiota throughout the study, as expected from previous longitudinal studies in humans [47,48]. Samples from the same individual were significantly more similar than to samples from other individuals regardless of timepoint (Figure 3a, R^2^ = 0.67, *p* < 0.001), however pairwise comparisons between participants did not reach significance. No significant relationship was seen between samples taken at the same timepoint from different participants. Notably, the microbial community profiles of participant 1 and 2 were each clustered distinctly away from other participants, corresponding with their distinct separations from other participants in diet at the level of food items (Figure 2c).

A post-competition temporal shift was observed in terms of the within- and between-sample diversities (Figure 3). The PRE8 and PRE1 microbial communities of each individual were more similar than to the POST4 community in four out of five participants, as shown with the weighted UniFrac between-sample diversity measure (Figure 3b). The POST4 microbial communities also had the lowest within-sample diversity in four out of five participants as measured by the inverse Simpson index (Figure 3c). Thus, in all participants the post-competition regime exerted a discernible impact on the microbiota.

Across all participants, the majority of the microbial community (55–85%) were assigned to the phylum *Firmicutes* (Figure 3d,e). The *Verrucomicrobia* and *Actinobacteria* phyla showed between-participant variations in abundance that were resilient to timepoint (Figure 3d), contributing to the individuality observed in principle coordinates analysis (Figure 3a).

In summary, individualised gut microbial communities were maintained across timepoints. There is evidence that the diet and/or exercise changes associated with the transition between pre- and post-competition may impact an individual’s microbiota, as the POST4 timepoint was an outlier across participants. The POST4 timepoint was not associated with changes in specific taxa but had a less complex microbial community structure with reduced richness and evenness.

### 3.4. Circulating Metabolite Profiles Reflect Individuality and Distinguishes between Pre- and Post-Competition

Next, we investigated the serum metabolic profiles of participants in a fasted and exercise-abstained state. Out of 127 detected metabolites, 9 were significant by timepoint, as measured by the nonparametric Kruskal–Wallis test (Table S3). The subset of metabolites that differed by timepoint was then examined to determine which timepoint was the outlier.

**Figure 4:**
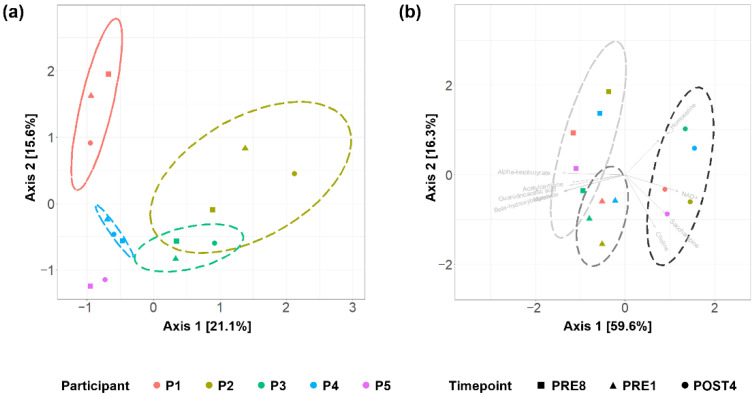
Circulating metabolites profiles differentiated by timepoint: Principal component analysis of (**a**) all metabolites; (**b**) metabolites significant by timepoint (Table S3); Ellipses indicate 95% confidence intervals of samples from each participant or timepoint; Vectors indicate the contribution of each metabolite to serum metabolite profiles; The PRE1 sample for P5 did not pass LC-MS quality control and was excluded from analysis.

Each participant maintained a unique metabolite profile over time when all metabolites were analysed (Figure 4a). Visualisation of the subset of metabolites that differed significantly by timepoint showed that the POST4 profiles were segregated from the PRE8 and PRE1 profiles (Figure 4b). The pre-competition profiles were characterised by elevated levels of acetylcarnitine, β-hydroxybutyrate, α-ketobutyrate, malonate, and guanidinoacetic acid, while the POST4 profiles were characterised by elevated levels of NAD+, saccharopine and choline (Figure 4b, Table S3). Measured metabolites that are known to be metabolised by the gut microbiota did not differ significantly by participant or by timepoint.

In summary, the circulating metabolite profiles showed a strong pattern of individuality, but a subset of metabolites showed distinct patterns between pre- and post-bodybuilding competition. Although the transition between before and after the competition shifted both the gut microbiota and the metabolome, there was no evidence that the two were interdependent.

## 4. Discussion

Bodybuilders use both dietary and exercise strategies to alter their body composition in preparation for a competition [27,28,29]. In our pilot study, all participants were successful in reducing fat mass while preserving lean mass during the pre-competition period. The two participants that had the least reduction of resistance training at the PRE1 timepoint relative to the baseline (PRE8) had the greatest preservation of lean mass. Comparatively, the participants with the greatest reduction in dietary energy intake during pre-competition had a greater loss of fat mass. However, the variations in bodybuilding outcomes could not be fully explained by exercise and diet. Therefore, we examined the responses of the gut microbiota and circulating metabolome across participants to identify common patterns of change in microbial and host metabolism that may have contributed to bodybuilding outcomes.

Diet drives microbial responses through mechanisms operating at multiple levels. The greatest difference in microbial community composition in our study was observed between individuals. Food choice plays a major role in shaping the microbial community as distinct chemical structures of food drive selection for specific microbes that can break down those compounds [11]. Correspondingly, the two participants with the most distinct diets at the level of food items compared to the rest of the cohort also had more distinct microbial profiles. At higher dietary levels, participants showed more similarities. The increase in dietary energy intake at post-competition compared to pre-competition corresponded with the lowest microbial within-sample diversities in an individual. Similarly, a study of obese and overweight individuals found that an energy-restricted dietary intervention increased the gut microbial gene diversity [49]. These findings suggest that changes in dietary energy intake can predict changes of microbial community complexity. At the level of dietary macronutrient distribution, there was a common shift from a high protein, low carbohydrate distribution during pre-competition to a more macronutrient-balanced diet post-competition. This macronutrient shift coincided with the greatest changes in between-sample microbial diversity within an individual. Notably, similar changes in microbial diversity in response to dietary energy density and macronutrient distribution were reported in a study of mice fed controlled diets comprising refined ingredients [31]. These results indicate that despite differences in the gut microbial community composition between individuals, the within-sample and between-sample diversity of the microbiota can be predictably modulated by diet. However, although detailed diet data was available in this study, a major limitation was that current food databases do not have an accurate analysis of dietary fibre content, especially resistant starch [50]. Dietary fibre encompasses a diverse range of substances, resulting in different definitions and measurement methods adopted over time [51,52]. This inconsistency has a large impact on diet-microbiota analyses as resistant starch degradation is highly specific to bacteria at the strain level [53], and degraded resistant starch products support the growth of a larger microbial community [54]. Therefore, better characterisation of dietary fibre in future studies is necessary to understand diet-microbiome relationships.

The overall circulating metabolite profiles showed stable interindividuality over the experimental period. There was no evidence of changes relating to microbial metabolism, but there were converging patterns across participants relating to lipid metabolism. The levels of acetylcarnitine and the ketone body β-hydroxybutyrate were elevated during pre-competition compared to post-competition. These metabolites are indicative of fatty acid oxidation in ketogenesis to generate energy when there is a lack of glucose supply, such as during exercise [55]. Multiple studies reported the greatest circulating metabolic changes within a few hours after exercise were related to lipid metabolism, with increases in medium- and long-chain fatty acids, ketone bodies, and fatty acid oxidation products, as recently reviewed [56]. In our study, participants did not exercise for 12 h prior to blood collection for metabolomic analysis. Thus, these observations suggest that the effects of long-term exercise on metabolism remain even at rest state. These common metabolic responses to exercise across individuals may have potential to provide insights into exercise outcomes.

The main limitation of this study is the small sample size, meaning that we were unable to perform correlation analysis between diet, exercise, gut microbes and serum metabolites. This is a common problem in human studies, where the high dimensionality of collected data results is often orders of magnitude larger than the number of samples available. The result is that identified correlations have a high chance of being spurious and not applicable outside the study population. Therefore, future studies would benefit by reducing the dimensionality of collected data, for instance, by computational methods such as machine learning [57], or biologically relevant methods such as guild-based analysis of the microbiome [58,59,60].

## 5. Conclusions

In conclusion, despite the small sample size, it was clear that the strongest pattern in the gut microbiota and circulating metabolite profiles was high interindividual variability. This individuality indicates that predicting the dynamics between diet, exercise, the gut microbiome and circulating metabolites would be more successful within one individual than across multiple individuals. Therefore, designing personalised diet and exercise regimes for both athletes and non-athletes would yield greater benefits than applying regimes based on generalised patterns from a population. This same recommendation has been proposed for both diet and microbiome data collection to improve studies investigating diet–microbiome relationships [61]. Similarly, to generate more useful data for modelling cardiometabolic disease risk and other health outcomes, we suggest longitudinal sampling of individuals undergoing different conditions rather than sampling a greater number of individuals cross-sectionally.

## Data Availability

The microbial raw sequence reads presented in this study are openly available in the National Center for Biotechnology Information (NCBI) Sequence Read Archive, accession numbers SRR10317043-SRR10317057.

## Supplementary Materials

The following supporting information can be downloaded at: https://www.mdpi.com/article/10.3390/metabo12100911/s1, Figure S1: Serves of food consumed as compared to the minimum recommended daily intake (MRDI); Figure S2: Principal component analysis of food items; Table S1: Body composition, exercise training, energy intake and macronutrient intake measured in the seven days prior to each time point; Table S2: Supplement intake in the seven days prior to each timepoint; Table S3: Circulating metabolites significantly different (*p* < 0.05) by timepoint.
