# Supplementary material for: Intersection of Diet and Exercise with the Gut Microbiome and Circulating Metabolites in Male Bodybuilders: A Pilot Study
Source: Metabolites. 2022 Sep 27;12(10):911. doi: 10.3390/metabo12100911 (PMC9608465; doi:10.3390/metabo12100911)
Supplement: Supplementary file 1 [file metabolites-12-00911-s001.zip › Supplementary Materials - Figure S1-2_rev2.pdf]

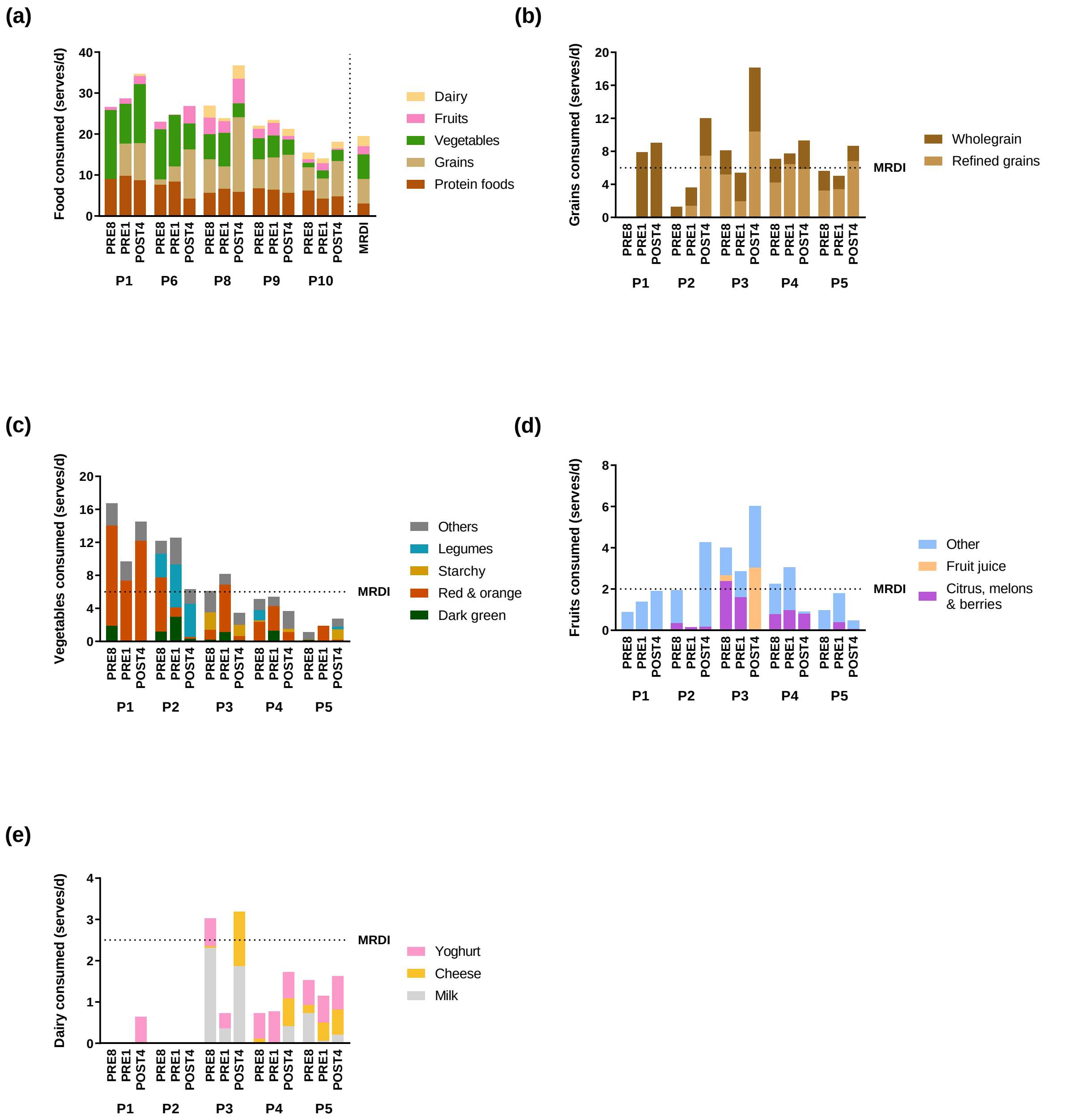

Figure S1: Serves of food consumed as compared to the minimum recommended daily intake (MRDI) [46].

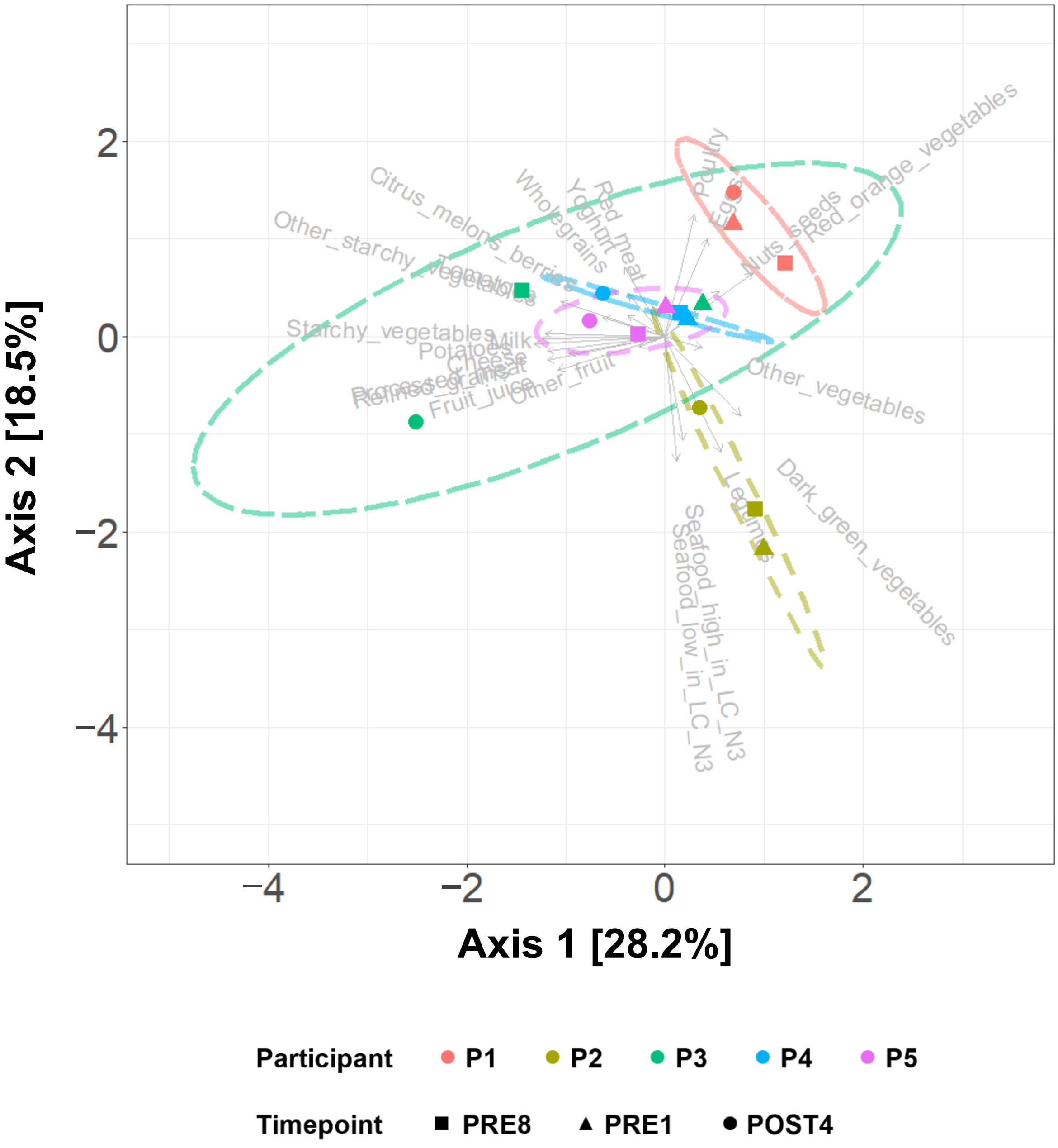

Figure S2: Principal component analysis of food items; Vectors indicate the contribution of each food item to the diet; Ellipses indicate 95% confidence intervals of samples from each participant
