# Supplementary material for: Intersection of Diet and Exercise with the Gut Microbiome and Circulating Metabolites in Male Bodybuilders: A Pilot Study
Source: Metabolites. 2022 Sep 27;12(10):911. doi: 10.3390/metabo12100911 (PMC9608465; doi:10.3390/metabo12100911)
Supplement: Supplementary file 1 [file metabolites-12-00911-s001.zip › Supplementary Materials - Table S1-S3_rev2.pdf]

Table S1: Body composition, exercise training, energy intake and macronutrient intake measured in the seven days prior to each time point.

Participant	Timepoint	Lean mass (kg)	Fat mass (kg)	Resistance training (kg/week)	Aerobic training (minutes/week)	Energy (kJ/kg)	Protein (g/kg)	Fat (g/kg)	Carbohydrate (g/kg)
**P1**	**PRE8**	73.5	5.39	87832	27	129	3.29	1.08	1.71
	**PRE1**	71.7	5.04	34346	0	184	4.42	1.38	3.19
	**POST4**	74.1	6.98	44884	0	150	3.07	1.04	3.28
**P2**	**PRE8**	55.3	11.51	42010	80	84	2.03	0.40	1.79
	**PRE1**	55.1	10.21	48369	150	90	2.39	0.28	1.91
	**POST4**	56.2	14.31	23341	0	128	0.91	0.77	4.62
**P3**	**PRE8**	74.3	7.74	51297	124	179	2.74	1.41	4.44
	**PRE1**	73.9	4.44	38950	16	132	2.55	1.00	2.61
	**POST4**	75.9	7.45	29545	0	263	3.17	2.22	7.27
**P4**	**PRE8**	70.4	6.68	68370	112	137	2.63	0.69	3.55
	**PRE1**	69.0	4.40	32970	40	118	2.14	0.59	3.26
	**POST4**	71.5	6.47	100441	20	149	2.72	0.93	3.79
**P5**	**PRE8**	63.3	3.64	89818	200	119	2.64	0.97	2.09
	**PRE1**	61.6	2.76	68168	190	105	1.92	0.61	2.83
	**POST4**	64.8	3.87	60792	0	157	2.58	1.42	3.63

Table S2: Supplement intake in the seven days prior to each timepoint.

Participant	Timepoint	Multi-supplements						Protein / amino acids						Vitamins / minerals							Others			
		Bulk Nutrients Green Fusion (g)	Giant Rush Pre-Workout (g)	Cellucor C4 Pre-Workout (g)	P2P Intra Workout (g)	Genetix ShredFX (g)	Genetix Revive (g)	Whey protein (g)	Nubreed Helix BCAA (g)	BCAA (g)	Glutamine (g)	Aspartic acid (g)	Genetix Beta alanine (g)	Multivitamin (tablet)	Vitamin C (tablet)	Vitamin D (mg)	Vitamin D3 (tablet)	Magnesium (tablet)	Magnesium (g)	Zinc (mg)	Acetyl carnitine (g)	Glucosamine (tablet)	Fish oil (g)	Creatine (g)
**P1**	**PRE8**	35							105	70	70	14		7							14		14	35
	**PRE1**	35								70	70			7									21	35
	**POST4**									70	70			7									21	35
**P2**	**PRE8**																							
	**PRE1**																							
	**POST4**																							
**P3**	**PRE8**																	4					21	15
	**PRE1**		5.2	6				30			16							4						20
	**POST4**			15																				
**P4**	**PRE8**				60			150							1									10
	**PRE1**					28	84						28			1.05			4.2	280			63	35
	**POST4**							150																
**P5**	**PRE8**													7								7		35
	**PRE1**													7			7					7	56	35
	**POST4**																							

Table S3: Circulating metabolites significantly different ($p < 0.05$) by timepoint.

**Super pathway**	**Pathway**	**Metabolite**	**KEGG**	**HMDB**	**Significant difference by timepoint (p-value)***	**Mean fold change (post-competition / pre-competition)**
Amino acid	Creatine metabolism	Guanidinoacetic acid	C00581	HMDB00128	0.003	0.3
	Glycine, serine, threonine metabolism	L-Homoserine	C00263	HMDB00719	0.024	1.2
		α-Ketobutyrate	C00109	HMDB00005	0.001	0.4
	Lysine metabolism	Saccharopine	-	HMDB00279	0.004	1.3
Cofactor	Nicotinate and nicotinamide metabolism	NAD^+	C00003	HMDB00902	0.001	1.2
Lipid	Fatty acid metabolism	Acetylcarnitine	C02571	HMDB00201	0.007	0.8
	Fatty acid synthesis	Malonate	C00383	HMDB00691	0.003	0.2
	Ketone body	β-Hydroxybutyrate	C01089	HMDB00357	0.003	0.2
	Phospholipid metabolism	Choline	C00114	HMDB00925	0.035	1.2

*Kruskal-Wallis test
